# The APE2 Exonuclease Is a Client of the Hsp70–Hsp90 Axis in Yeast and Mammalian Cells

**DOI:** 10.3390/biom12070864

**Published:** 2022-06-21

**Authors:** Siddhi Omkar, Tasaduq H. Wani, Bo Zheng, Megan M. Mitchem, Andrew W. Truman

**Affiliations:** Department of Biological Sciences, The University of North Carolina at Charlotte, Charlotte, NC 28223, USA; twani@uncc.edu (T.H.W.); bzheng1@uncc.edu (B.Z.); mmitch92@uncc.edu (M.M.M.)

**Keywords:** Hsp70, Hsp90, APE2, Apn2, cancer, chaperone inhibition

## Abstract

Molecular chaperones such as Hsp70 and Hsp90 help fold and activate proteins in important signal transduction pathways that include DNA damage response (DDR). Previous studies have suggested that the levels of the mammalian APE2 exonuclease, a protein critical for DNA repair, may be dependent on chaperone activity. In this study, we demonstrate that the budding yeast Apn2 exonuclease interacts with molecular chaperones Ssa1 and Hsp82 and the co-chaperone Ydj1. Although Apn2 does not display a binding preference for any specific cytosolic Hsp70 or Hsp90 paralog, Ssa1 is unable to support Apn2 stability when present as the sole Ssa in the cell. Demonstrating conservation of this mechanism, the exonuclease APE2 also binds to Hsp70 and Hsp90 in mammalian cells. Inhibition of chaperone function via specific small molecule inhibitors results in a rapid loss of APE2 in a range of cancer cell lines. Taken together, these data identify APE2 and Apn2 as clients of the chaperone system in yeast and mammalian cells and suggest that chaperone inhibition may form the basis of novel anticancer therapies that target APE2-mediated processes.

## 1. Introduction

The well-conserved Hsp70 and Hsp90 molecular chaperones are critical for the folding, maturation and activity of a large number of “client” proteins [1]. Client proteins are found in diverse cellular pathways, and consequently, chaperones support the maintenance of apoptotic signaling, angiogenesis, autophagy, senescence [1,2,3]. Although prokaryotes possess a single prototypical Hsp70 and Hsp90 (DnaK and HtpG, respectively), eukaryotes possess several paralogs that differ in their subcellular localization and expression profile [4,5,6]. In budding yeast, the main cytosolic forms of Hsp70 are Ssa1–4, which arose from multiple gene duplication events. Ssa1 and 2 are constitutively expressed at high levels, whereas Ssa3 and 4 are highly heat inducible [7,8,9]. The Ssa paralogs are semi-redundant, evidenced by the fact that yeast remain viable as long as they have one paralog expressed at constitutively high levels [7,8,9]. Despite their relatedness, recent studies suggest that the Ssa paralogs have slightly different client binding profiles [4]. Similarly, humans encode 13 isoforms of Hsp70s from a multigene family with major cytosolic paralogs being HspA8 (constitutive) and HspA1A/HspA1L (inducible) [10,11,12]. Hsp90 also exists in various forms in cells. In mammalian cells, the inducible Hsp90a and constitutively expressed Hsp90b are the major species in the cytosol, equivalent to yeast Hsp82 and Hsc82, respectively [5,13]. A major stress that cells must deal with to survive are challenges to genome integrity in the form of DNA damage [14]. The sensing of DNA damage and its repair are mediated by an array of proteins that together form the DNA damage response (DDR) pathway [15]. While chaperones support many key signal transduction pathways in the cell, evidence is building to support a particularly critical role for chaperones in the detection and repair of DNA damage. Hsp70 and Hsp90 support DDR by activating and stabilizing a huge number of DDR proteins including p53, CHK1, FANCA, FANCD2, BRCA1/2, MRN and RNR complexes [16,17,18]. A common type of DNA damage is the loss of a base from genomic DNA, known as apurinic/apyrimidinic (AP) sites. The repair of such sites involves the recruitment of the related APE1 and APE2 exonucleases (Apn1 and Apn2 in yeast) [19,20,21,22,23,24]. Although APE1 and APE2 display functional overlap, APE2 possesses an extra C-terminal domain that is absent in APE1 and lacks any redox activity [22]. A recent study examined global protein abundance and epigenetic changes in response to Hsp90 inhibition. Several DDR proteins were among those found to decrease upon ganetespib and AUY922 treatment, including XRCC1, XPC and APE2 [25]. While APE1 becomes associated with Hsp70 during DNA repair to augment endonuclease activity, no such mechanistic connection between chaperones and APE2 has been identified [26]. In this study, we demonstrate a novel interaction between APE2/Apn2 and the Hsp70–Hsp90 system in yeast and mammalian cells. Although there appears to be no preference for which Hsp90 or Hsp70 paralog APE2/Apn2 bind, yeast Apn2 is destabilized in yeast lacking Ssa2, 3 and 4. Inhibition of Hsp90 via ganetespib or Hsp70 via JG-98 triggered a surprisingly rapid reduction of APE2 in a range of cancer cell lines. Understanding the intricacies of chaperone–endonuclease interactions could lead to more targeted and less toxic cancer therapeutics that exploit the genomic instability often seen in tumor cells.

## 2. Materials and Methods

### 2.1. Yeast Strains and Growth Conditions

Yeast cultures were grown in either YPD (1% yeast extract) US Biological Life Sciences, Swampscott, MA, USA, 2% glucose (VWR, Radnor, PA, USA), 2% peptone (Thermo Fisher Scientific, Waltham, MA, USA) or in SD (0.67% yeast nitrogen base without amino acids and carbohydrates (US Biological Life Sciences), 2% glucose), supplemented with the appropriate nutrients to select for plasmids and tagged genes. *Escherichia coli* DH5α was used to propagate all plasmids. *E. coli* cells were cultured in Luria broth medium (1% Bacto tryptone, 0.5% Bacto yeast extract, 1% NaCl) and transformed to ampicillin resistance by standard methods. Hsp70 isoform plasmids were transformed into yeast strain *ssa1–4*∆ [27] using PEG/lithium acetate. After restreaking onto media lacking leucine, transformants were streaked again onto media lacking leucine and containing 5-fluoro-orotic acid (5-FOA) (US Biological Life Sciences), resulting in yeast that expressed Hsp70 paralogs as the sole cytoplasmic Hsp70 in the cell. For a full description of yeast strains see Table 1 and for plasmids see Table 2.

### 2.2. Purification of HA-Tagged Apn2 from Yeast

The protocol followed for HA-IP was taken from [28] with slight modifications. Cells transformed with control pRS316 plasmid or the plasmid-expressing HA-tagged Apn2 [26] were grown overnight in SD-URA media and then re-inoculated into a larger culture of selectable media and grown to an OD_600_ of 0.800. Cells were harvested, and HA-tagged proteins were isolated as follows. Protein was extracted via bead beating in 500 µL protein extraction buffer (50 mM Na-phosphate pH 8.0, 300 mM NaCl, 0.01% Tween-20). Then, 1000 µg of protein extract was incubated with 25 µL anti-HA magnetic beads (Thermo Fisher Scientific) at 30 °C for 30 min. Anti-HA beads were collected by magnet and then washed 3 times with TBS-T and 2 times with protein extraction buffer. After the final wash, the buffer was aspirated, and beads were incubated with 75 µL protein extraction buffer, and 25 µL 5× SDS-PAGE sample buffer sample was denatured for 5 min at 95 °C and boiled for 10–15 min. Next, the beads were collected via magnet, and the supernatant-containing purified HA-Apn2 was transferred to a fresh tube. Then, 20 µL of each sample was analyzed on SDS-PAGE.

### 2.3. Mammalian Cell Culture and Drug Treatment

The protocol used for transfection and drug treatment was taken from [22] with slight modifications. HEK293T cells were cultured in Dulbecco’s modified Eagle’s minimal essential medium (DMEM; Invitrogen, Carlsbad, CA, USA) supplemented with 10% fetal bovine serum (FBS; Invitrogen), 100 U/mL penicillin (Invitrogen) and 100 µg/mL streptomycin (Invitrogen). L-GlutaMAX nutrient mixture (Gibco, Waltham, MA, USA, Cat#31765-035) (10% FBS, 100 units of penicillin and 100 units of streptomycin) was used to culture PC3, RPMI 1640 based medium (10% FBS, 100 units of penicillin and 100 units of streptomycin, 1% L-GlutaMAX-I) for LNCaP and DMEM-based medium (10% FBS, 100 units of penicillin and 100 units of streptomycin, 1% L-GlutaMAX-I) for MCF7. All cell lines were incubated at 37 °C in a 5% CO_2_ containing atmosphere. Cells were seeded in 6-well plates at 1 × 10^6^/2 mL per well one day prior to transfection. Cells were transfected by APE2 expression plasmid pcDNA-APE2-HA-BCP [29] with Lipofectamine3000 transfection kit (Invitrogen, Cat#L3000-015), and 2.5 μg of DNA and 7.5 μL of Lipofectamine3000 were used for each well. Briefly, diluted Lipofectamine3000 and DNA plus P3000 with Opti-MEM I (Gibco, Cat#31985-070) were mixed and incubated at room temperature for 15 min and then added to cell culture dropwise. The cells were treated for 0, 2, 4, 8 and 16 h post 48 h transfection with 10 μM JG-98, which is a Hsp70 inhibitor or 10 μM ganetespib (STA-9090, Selleckchem, Houston, TX, USA, Cat#S1159) for Hsp90 inhibition.

### 2.4. Transfections and Co-Immunoprecipitation in Mammalian Cells

The protocol used for transfection and drug treatment was adapted from [28] with slight modifications. HEK293T cells or specific cancer cells such as PC3, LNCaP and MCF7 were either untransfected (mock) or transfected with plasmids for expression of HA-tagged and/or V5-tagged proteins for constitutive HSPA8 and inducible HSPA1L and HSPA1A using Lipofectamine 3000 (Thermo Fisher Scientific). After 48 h, the cells were washed with 1X PBS, and total cell extract was prepared from the cells using M-PER (Thermo Fisher Scientific) containing EDTA-free protease and phosphatase inhibitor cocktail (Thermo Fisher Scientific) according to the manufacturer’s recommended protocol. Protein was quantitated using the Bradford Assay. HA-tagged proteins were purified as follows. First, 200 µg of protein extract was incubated with 25 µL anti-HA magnetic beads (Thermo Fisher Scientific) at 30 °C for 30 min. Anti-HA beads were collected by magnet and then washed 3 times with TBS-T and 2 times with protein extraction buffer. After the final wash, the buffer was aspirated, and beads were incubated with 75 µL protein extraction buffer, and 25 µL 5× SDS-PAGE sample buffer sample was denatured for 5 min at 95 °C and boiled for 10–15 min. Next, the beads were collected via magnet, and the supernatant-containing purified HA-APE2 was transferred to a fresh tube. Finally, 20 µL of each sample was analyzed on SDS-PAGE.

### 2.5. Western Blotting

First, 20 µg of protein was separated by 4–12% NuPAGE SDS-PAGE (Thermo Fisher Scientific). Proteins were detected using the following antibodies; anti-HA tag (Thermo Fisher Scientific), Anti-FLAG tag (Sigma-Aldrich, St. Louis, MO, USA, USA #F1365), anti-PGK (Thermo Fisher Scientific, #MA5-15738), anti-Ydj1 (Stressmarq Biosciences Inc., Victoria, BC, Canada, #SMC-166D), anti-HDJ2 (Thermo Fisher Scientific, #MA512748). Blots were imaged on a ChemiDoc MP imaging system (Bio-Rad, Hercules, CA, USA). After treatment with Super Signal West Pico Chemiluminescent Substrate (GE Healthcare, Piscataway, NJ, USA). Blots were stripped and reprobed with the relevant antibodies using Restore Western Blot Stripping Buffer (Thermo Fisher Scientific).

## 3. Results

### 3.1. Apn2 Interacts with Ydj1, Hsp82 and Ssa1 in Yeast

**Figure 1:**
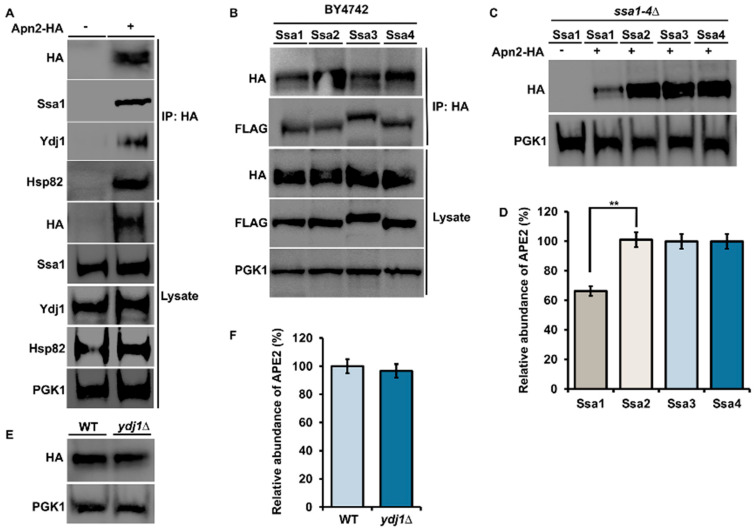
Apn2 interacts with Hsp82, Hsp70 and Ydj1 in yeast. (**A**) Yeast cells expressing Apn-HA were grown to mid-log phase at 30 °C. Lysate from these cells were analyzed by Western blotting with an anti-HA, anti-Ssa1, anti-Ydj1 and anti-Hsp82 antibody. Pgk1 was used as a loading control. Immunoprecipitation was performed using anti-HA magnetic beads, and the interaction was studied. (**B**) WT cells were co-transformed with Apn2-HA and individual Ssa isoforms. Yeast cells were grown to mid-log phase at 30 °C. Lysates were analyzed by Western blotting with HA and FLAG specific antibody. Immunoprecipitation was performed using anti-HA magnetic beads, and interaction between FLAG-Ssa and Apn2-HA was checked using anti-HA and anti-FLAG antibodies on Western blot. (**C**) Yeast expressing the indicated FLAG-Ssa (on a constitutive promoter) in a *ssa1–4*∆ background transformed with Apn2-HA were grown to mid-log phase at 30 °C. Lysates were analyzed by Western blotting with HA- and FLAG-specific antibodies. (**D**) Relative abundance of Apn2-HA was quantitated by taking the ratio of Apn2-HA/PGK1. Data are the mean and SD of three replicate experiments and compared to Ssa2 (** *p* < 0.001) (**E**) WT BY4742 and Ydj1∆ cells, were transformed with HA-Apn2 plasmid. Transformants were grown to mid-log phase at 30 °C. Lysate from these cells was analyzed by Western blotting with an anti-HA and anti-Ydj1 antibody. (**F**) Relative abundance of Apn2-HA was quantitated by taking the ratio of Apn2-HA/PGK1. Data are the mean and SD of three replicate experiments and compared to WT.

Previous studies suggested that inhibition of Hsp90 may lead to loss of APE2 in bladder cancer [25]. To determine whether there was a connection between yeast APE2 (Apn2) and chaperones, we purified HA-tagged Apn2 from yeast and probed the complex with anti-HA, anti-Hsp82, anti-Ssa1, and anti-Ydj1 antibodies. We observed a clear association with Ssa1, Hsp82 and Ydj1 (Figure 1A). There are four cytosolic Hsp70s in yeast, Ssa1, 2, 3 and 4, which are highly similar to the amino acid sequence that arose from multiple yeast gene duplication events [4]. While these paralogs have clear functional overlap, they also display differential client preferences [4]. To determine whether all Ssa paralogs can interact with Apn2, we performed co-immunoprecipitation experiments in WT BY4742 yeast cells (Table 1) expressing plasmids-HA-Apn2 and exogenous Flag-Ssa1, 2, 3 or 4 (Figure 1B). In this context, Apn2 bound equally to all Ssa paralogs (Figure 1B). To query whether all four Ssa paralogs could support Apn2 stability, we examined the levels of constitutively expressed HA-Apn2 in *ssa1–4*∆ yeast, expressing only one of the four Ssa proteins (Table 1). The levels of Apn2 were significantly decreased in yeast-expressing Ssa1 as the sole Ssa paralog in the cell (Figure 1C,D). Co-chaperones of Hsp70 play an important role in regulating chaperone activity and specificity [30]. We wondered whether Ydj1, a major co-chaperone of Ssa1–4, may support Apn2 levels in a similar way to its chaperoning of the ribonucleotide complex [28]. To test this possibility, we compared the abundance of Apn2 in WT yeast and those lacking Ydj1 (Table 1). In contrast to the regulation of RNR, the lack of Ydj1 had minimal impact on Ape2 levels (Figure 1E,F).

### 3.2. Apn2 Interacts with Both Hsp82 and Is a Client of Hsp90 in Yeast

**Figure 2:**
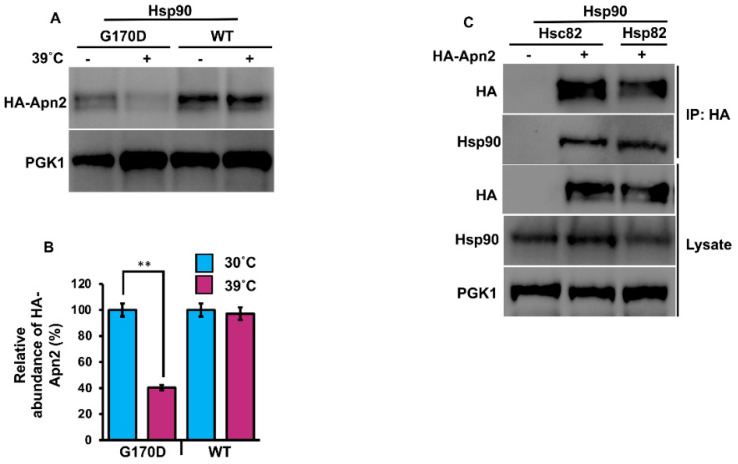
Apn2 interacts with Hsc82 and Hsp82. (**A**) Yeast G170D and P82a cells expressing Apn2-HA were grown to mid-log phase at 30 °C. Cells were stressed at 39 °C, and lysates from unstressed and heat shocked cells were analyzed for Apn2 levels using Western blot with anti-HA antibodies. Pgk1 was used as a loading control. (**B**) Relative abundance of Apn2-HA was quantitated by taking the ratio of Apn2-HA/PGK. Data are the mean and SD of three replicate experiments, and further, unstressed cells were compared to heat shocked cells (** *p* < 0.001). (**C**) Hsc82-Glu and Hsp82-His yeast cells were transformed with Apn2-HA. Cells were grown to mid-log phase at 30 °C. Lysate from these cells was analyzed by SDS-PAGE and Western blotting using anti-HA and yeast anti-Hsc82-specific antibodies. Pgk1 was used as a loading control. Immunoprecipitation was performed using anti-HA magnetic beads, and the interaction was studied.

Our previous results suggested that Apn2 may also be a direct client of Hsp90. To test this hypothesis, we examined Apn2 in yeast expressing a well-characterized temperature sensitive point mutation in Hsp90 [31]. Cells expressing HA-Apn2 in either Hsp82^G170D^ (Table 1) or WT (Table 1) were grown at 25 °C until early mid-log phase and were split into two flasks, one of which was shifted to 39 °C. Cells were lysed after 90 min, and HA-Apn2 levels were examined by Western blot. Incubation at 39 °C caused a significant decrease in HA-Apn2 levels in Hsp82^G170D^ cells, while HA-Apn2 levels remained unchanged in WT cells, confirming Apn2 as a client of Hsp90 (Figure 2A). There are two Hsp90 paralogs in yeast, the heat-inducible Hsp82 and constitutive Hsc82 (Table 1). To assess Apn2 binding preferences for the two Hsp90s, we purified Apn2 from yeast expressing tagged versions of Hsp82 or Hsc82 using anti-HA magnetic beads. Consistent with our results in Figure 1B (above), the binding of Apn2 was equal to both heat-inducible Hsp82 and constitutive Hsc82 (Figure 2C).

### 3.3. Mammalian APE2 Interacts with the Hsp90–Hsp70 Chaperone System

**Figure 3:**
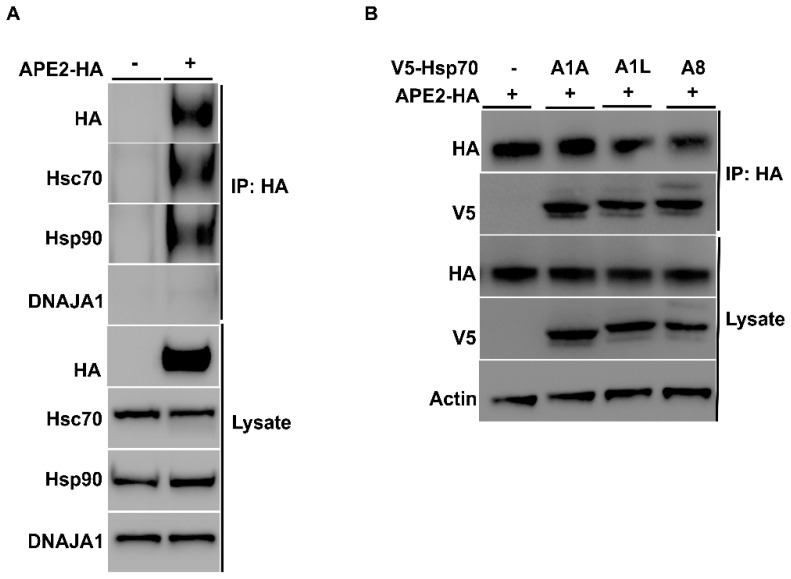
Mammalian APE2 interacts with the Hsp90–Hsp70 chaperone system. (**A**) HEK293 cells were grown to mid-confluence and were transfected with a construct expressing HA-APE2 from a constitutive CMV promoter. After 48 h, cells were lysed, and APE2 complexes were purified using anti-HA-magnetic beads. Lysates from these cells were analyzed by SDS-PAGE and Western blotting using anti-HA, anti-Hsp70, anti-DNAJA1 and anti-Hsp90 specific antibodies. Beta-actin was used as a loading control. Immunoprecipitation was performed using HA beads, and the interaction was studied. (**B**) HEK293 cells were co-transfected with V5-tagged Hsp70 and APE2-HA. Immunoprecipitation was performed using anti-HA-magnetic beads, and the interaction was studied using anti-V5 and anti-HA antibody.

Mammalian APE2 plays a variety of roles in key cellular processes involving the response to a multitude of stressors, including DNA single- and double-strand breaks, base excision repair, and oxidative stress, leading to the activation of DDR complexes and pathways, including ATR and Chk1 [16,18]. The abundance of several DDR proteins, including APE2, decreased in bladder cancer cells treated with Hsp90 inhibitors [25]. To determine if there was a physical interaction between chaperones and APE2, we took a similar approach to that of Figure 1. HEK293 cells were grown to mid-confluence and were transfected with a construct expressing HA-APE2 (Table 2). After 48 h, cells were lysed, and APE2 complexes were purified using anti-HA magnetic beads. SDS-PAGE analysis and Western blotting of APE2 complexes revealed the presence of Hsp70 and Hsp90, which were not observed in the immunoprecipitation from cells lacking HA-APE2 (Figure 3A). Despite the robust interaction of APE2 with the chaperones, the major DNAJA1 co-chaperone was not observed in the APE2 complex (Figure 3A).

There are a variety of Hsp70 family members expressed in mammalian cells. Although they are highly conserved, they vary in their client selectivity, cellular localization and expression pattern in tissues [11,12,32]. Our previous results suggested that APE2 interacts with HSPA8, the major constitutively expressed isoform of Hsp70 in cells. To determine whether APE2 might be able to bind other HSPA family members, we co-transfected HEK293 cells with plasmids (Table 2) expressing HA-APE2 and V5-tagged HSPA family members that included inducible HSPA1A, HSPA1L and non-inducible HSPA8. After 48 h, we purified HA-APE2 from these cells and subjected the complex to analysis by SDS-PAGE/Western blotting (Figure 3B). Consistent with our results in yeast, APE2 binding was observed between both the constitutive and heat-inducible expressed HSPs in mammalian cells (Figure 3B).

**Figure 4:**
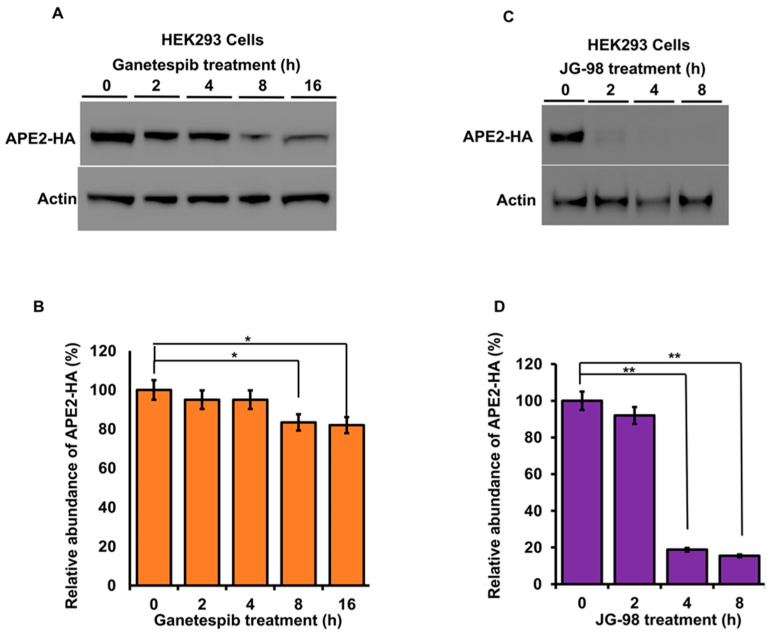
Inhibition of Hsp90 or Hsp70 promote a rapid reduction in APE2 levels. (**A**) HEK293 cells expressing HA-APE2 were treated with either an inhibitor of Hsp90 (ganetespib) or (**C**) Hsp70 (JG-98). Cells were harvested at the indicated time points, and APE2 abundance was determined by SDS-PAGE and Western blotting using anti-HA antibody. Beta-actin was used as a loading control. (**B**,**D**) The relative abundance of APE2-HA was quantitated by taking the ratio of Apn2-HA/Beta-actin from 3 replicate experiments and compared to untreated HEK293 cells. Data are the mean and SD of three replicate experiments and are compared to untreated. Statistical significance is indicated as (** *p* < 0.001) (* *p* < 0.05).

**Figure 5:**
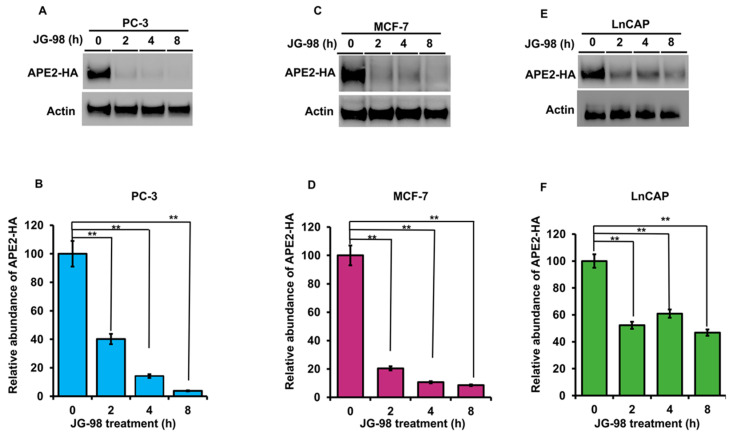
Stability of APE2 in a range of cancer cell lines is dependent on Hsp90 and Hsp70 function. (**A**) PC3 (**C**) MCF7 and (**E**) LnCAP cells expressing HA-APE2 were treated with an inhibitor of Hsp70 (JG-98). Cells were harvested at the indicated time points, and APE2 abundance was determined by SDS-PAGE and Western blotting using anti-HA antibody. Beta-actin was used as a loading control. (**B**,**D**,**F**) The relative abundance of APE2-HA was quantitated by taking the ratio of APE2-HA/Beta-actin. Data are the mean and SD of three replicate experiments and are compared to untreated (** *p* < 0.001).

**Figure 6:**
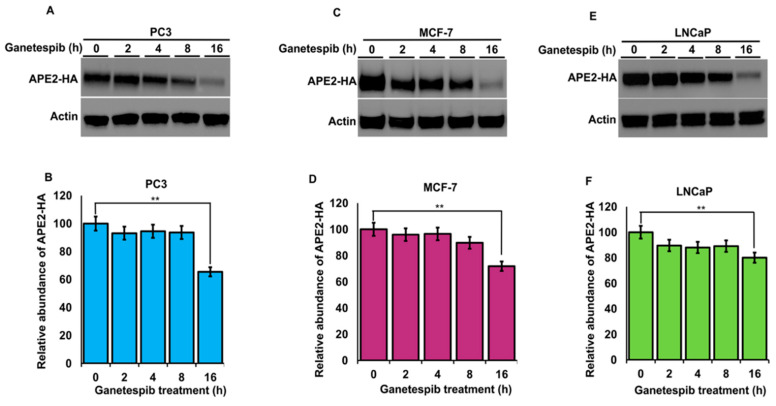
Stability of APE2 in a range of cancer cell lines is dependent on Hsp90 function. (**A**) PC3 (**C**) MCF7 and (**E**) LnCAP cells expressing HA-APE2 were treated with an inhibitor of Hsp90 (ganetespib). Cells were harvested at the indicated time points, and APE2 abundance was determined by SDS-PAGE and Western blotting. Beta-actin was used as a loading control. (**B**,**D**,**F**) The relative abundance of APE2-HA was quantitated by taking the ratio of APE2-HA/Beta-actin. Data are the mean and SD of three replicate experiments and are compared to untreated (** *p* < 0.001).

The stability of APE2 in epithelial cells is dependent on Hsp70 and Hsp90 function. Molecular chaperones regulate the folding, maturation and stability of their client proteins [33]. Our previous results implied that APE2 may be a bona fide client of the Hsp90–Hsp70 system. To examine this possibility, we assessed the impact of chaperone inhibition on APE2 abundance. HEK293 cells expressing HA-APE2 were treated with either an inhibitor of Hsp90 (ganetespib) or Hsp70 (JG-98). Cells were harvested at the indicated time points, and APE2 abundance was determined by Western blotting. HEK293 cells treated with ganetespib showed a decrease in APE2 abundance after only 8 h of treatment (Figure 4A). Even more impressive was the rapid decrease in APE2 levels after only 2 h of treatment of JG-98 (Figure 4B). We queried whether this dependence extended to other cancer cell lines including breast cancer (MCF-7) as well as androgen-dependent and androgen-independent prostate cancer (LNCaP and PC-3, respectively). As with our previous experiments, these cell lines were treated with ganetespib, and APE2 levels were assessed through Western blotting at 2 h intervals. In the case of PC-3, MCF7 and LNCaP, the APE2 levels significantly decreased after 2 h of treatment of JG-98 (Figure 5A–F). To similarly understand whether Hsp70 contributed toward APE2 stability, we treated MCF-7, LNCaP and PC-3 cells with the Hsp90 inhibitor and measured APE2 abundance via Western blotting. APE2 levels started to decline significantly after 2 h of treatment with maximum inhibition seen at 16 h (Figure 6A–F).

## 4. Discussion

The ability of cells to repair and maintain their genome is critical for their survival. The response to DNA damage is highly complex and relies on several different signaling cascades comprising multiple proteins [14,15]. The Hsp70–Hsp90 chaperone system binds and regulates several important proteins in this process, including APE1 and P53. Recent efforts in understanding the role of chaperones in DDR have included large-scale proteomics analysis, such as that of Li et al., which examined the abundance of proteins in 5637 bladder cancer cells after treatment with the Hsp90 inhibitors ganetespib (STA9090), or luminespib (AUY-922) [25]. In that study, over 800 proteins were downregulated, including XRCC1, XPC, RAD50, 53BP1 and notably, APE2 [25]. In this study, we have identified a role for the Hsp70 and Hsp90 chaperones in regulating the activity of the APN2/Ape2 exonuclease in yeast and mammalian cells.

### 4.1. APE2 and Apn2 Display Binding Preferences for Chaperone and Co-Chaperone Paralogs

An unresolved question in chaperone biology is why cells express many highly similar chaperone proteins. In yeast, the four Ssa proteins are highly conserved with over 80% similarity in amino acids sequence [4]. Ssa1 and Ssa2 represent the major cytosolic Hsp70s present under basal conditions, while Ssa3 and Ssa4 are highly heat induced. Several studies have suggested that these chaperone paralogs have overlapping but unique interactomes [34]. Recently, work using the model substrate ribonucleotide reductase (RNR) showed a clear preference for this client in binding Ssa1 and Ssa2 [35]. Although Apn2 binds cytosolic Hsp70 and Hsp90 paralogs equally, cells expressing Ssa1 as their sole cytosolic Ssa1 are unable to support WT levels of Ape2 as depicted by compromised stability in Figure 1B,C. The difference in Apn2 abundance in Ssa2 vs. Ssa1-expressing yeast is particularly interesting considering how similar the two proteins are. However, previous studies have shown that even a single divergent amino acid between Ssa1 and Ssa2 can produce differences in their ability to modulate prion propagation and protein degradation [36]. A recent study observed a parallel defect in septin levels in Ssa1-expressing yeast [37]. Future research, possibly involving a comparative interactome study of Ssa proteins, may shed light on this issue [34].

Cells express a variety of co-chaperones that are critical for stimulation of chaperone ATPase activity and for loading clients onto chaperones for folding [3,30,38]. We show here that Apn2 co-purifies with Ydj1, a major Hsp70 co-chaperone (Figure 1A). In contrast to ribonucleotide reductase whose stability depends on Ydj1 function, loss of Ydj1 does not impact Apn2 stability [28]. It is possible that Apn2 stability in yeast is additionally regulated by other semi-redundant co-chaperones such as Sis1, which has similar yet distinct roles in the cell as Ydj1 [39,40,41,42]. This may also explain why in our studies, DNAJA1 the mammalian homologue of Ydj1 does not appear to interact with APE2 (Figure 3A). Going forward, it would be interesting to identify and understand the major co-chaperones responsible for regulating APE2 and Apn2 function in mammalian and yeast cells, respectively.

### 4.2. Novel Anticancer Strategies Based on Fine-Tuning Chaperone Function

Molecular chaperones are required for the stability and activity of many proteins, including oncoproteins that are critical for cancer progression [43,44,45,46]. Recently, APE2 has been revealed to be an important player in regulating genome integrity and cancer progression [20,22,23,29,47,48]. Our study suggests that targeting APE2 activity through inhibition of chaperone function may be a viable anticancer therapy. While in vitro studies such as those presented here clearly show the value of manipulating chaperone function, studies in vivo suggest that complete abolishment of Hsp70 or Hsp90 results in severe toxicity for patients [25,49]. Several alternative approaches to bypass the toxicity issue are currently being pursued [49,50,51]. The first has been to identify key co-chaperones that regulate oncogenic clients and to develop drugs that inhibit them, such as 116-9e and C-86 [52,53,54]. While DNAJA1 is not observed in complex with APE2, it is possible that drugs such as 116-9e and C-86 may have a broad enough specificity to be target regulatory co-chaperones of APE2 in cancer. An alternative method for fine-tuning of chaperones may be to manipulate their post-translational modifications (PTMs) [55,56,57]. Future studies examining the Hsp70/Hsp90-APE2 structure may allow for specific targeting of this interaction via small molecules that bind the interaction interface or alter critical PTMs required for chaperone–exonuclease interaction.

**Table 1:** Yeast strains used in this study.

Strain	Genotype	Reference/Source
yAT 685	Hsc82 (PP30-HSC82-GLU (MAT a, trp1-289, leu2-3112, his3-200, ura3-52, ade2-101, lys2-801, hsc82::KANMX4, hsp82::KANMX4 LEU2-GPD-HSC82-GLU)	[51]
yAT 686	Hsp82 PP30-HSP82-HIS (MAT a, trp1-289, leu2-3112, his3-200, ura3-52, ade2-101, lys2-801, hsc82::KANMX4, hsp82::KANMX4 LEU2-GPD-HSP82-HIS)	[51]
yAT01	P82a W303–1a hsc82::LEU2 hsp82::LEU2 HIS3-GPD-HSP82a	[31]
yAT05	G170D W303–1a hsc82::LEU2 hsp82::LEU2 HIS3-GPD-hsp82(G170D)a	[31]
yAT38	MATα S288c (BY4742) his3Δ1 leu2Δ0 lys2Δ0 ura3Δ0	Euroscarf
yAT414	MATa (MH272) ssa1∆::trp1 ssa2::HisG ssa3::HisG ssa4::HisG (ssa1–4) [YCPlac33 SSA1]	[27]
yAT28	MATα S288c (BY4742) ydj1∆::KanMX4	Euroscarf

**Table 2:** Plasmids used in this study.

Plasmids	Description	Reference
pNK229	GPD2-Apn2-HA	[18]
pAT778	pRS315PSsa2-Flag-SSA1 (LEU2)	Vector Builder
pAT779	pRS315PSsa2-Flag-SSA2 (LEU2)	Vector Builder
pAT780	pRS315PSsa2-Flag-SSA3 (LEU2)	Vector Builder
pAT781	pRS315PSsa2-Flag-SSA4 (LEU2)	Vector Builder
	APE2-HA	[23]
pAT758	HSPA1A-V5 pcDNA5/FRT/TO	Harm Kampinga
pAT759	HSPA1L-V5 pcDNA5/FRT/TO	Harm Kampinga
pAT763	HSPA8-V5 pcDNA5/FRT/TO	Harm Kampinga

Overall, this work identifies a new client of the Hsp70–Hsp90 axis, the Apn2/APE2 exonuclease. The rapid loss of APE2 in cancer cells upon inhibition of either Hsp90 or Hsp70 provides a path forward for novel therapies that jointly target chaperones and the DNA damage response.

## Data Availability

All data from this study can be found in the main manuscript.
